# Lower Extremity Muscle Performance and Foot Pressure in Patients Who Have Plantar Fasciitis with and without Flat Foot Posture

**DOI:** 10.3390/ijerph20010087

**Published:** 2022-12-21

**Authors:** Jin Hyuck Lee, Ki Hun Shin, Taek Sung Jung, Woo Young Jang

**Affiliations:** 1 Department of Sports Medical Center, Korea University College of Medicine, Seoul 02841, Republic of Korea; 2 TMX Limited, Seoul 06286, Republic of Korea; 3 Department of Orthopedic Surgery, Korea University College of Medicine, Seoul 02841, Republic of Korea

**Keywords:** plantar fasciitis, foot posture, muscle strength, muscle reaction time, foot pressure

## Abstract

Abnormal foot posture and poor muscle performance are potential causes of plantar fasciitis (PF). However, no study has compared the differences between lower extremity muscle performance and foot pressure in patients who have PF with and without abnormal foot postures. This study aimed to compare the differences in lower extremity muscle performance, such as in the hip, quadriceps, hamstring, and plantar flexor, and foot pressure in patients who have PF with and without flat foot postures. Seventy patients with plantar heel pain were enrolled (37 flat feet and 33 without flat feet). The hip muscle strength was measured using a handheld digital dynamometer. The strength and reaction time of the quadriceps, hamstring, and plantar flexor muscles were evaluated using an isokinetic device. Foot pressure parameters were assessed using pedobarography. The strength of the plantar flexor muscles was significantly lower (*p* = 0.008), while the reaction time of the plantar flexor muscles was significantly faster (*p* = 0.007) for the involved feet of PF patients with flat feet than in those without flat feet. This study confirmed the differences in muscle performance between patients who have PF with different foot postures. Therefore, clinicians and therapists should plan treatment considering the differences in these characteristics for the management of these patients.

## 1. Introduction

Plantar fasciitis (PF) is the most common cause of inferior heel pain among middle-aged adults [1,2]. It is aggravated by microtears of the plantar fascia from repeated stretching during walking or running [1,3]. PF may be adversely affected by factors, such as abnormal foot posture, lower extremity muscle weakness, Achilles tendon tightness, and being overweight [1,3,4]. Therefore, the accurate diagnosis, assessment, and management of PF by clinicians and podiatrists is important.

Abnormal foot posture, such as a flat foot, in patients with PF may lead to prolonged stretching of the plantar fascia due to loss of the foot arch, resulting in further damage to the plantar fascia [3,5,6]. On the other hand, in patients who have PF with a high arched foot posture, the plantar fascia may be damaged owing to poor shock absorption by reduced ground contact area [7,8]. Foot posture has traditionally been evaluated using plain radiography for foot alignment [9,10,11] and pressure platforms for foot pressure [5,9,12]. Particularly in patients with flat feet and PF, increased flattening of the foot during walking increases peak plantar pressure [12,13]. However, some studies [14,15,16] have failed to show a significant difference in foot posture between patients with PF and healthy controls. Similarly, Landorf et al. [17] reported that foot posture did not differ between patients with and those without PF. Thus, it is unclear whether abnormal foot posture, such as flat feet, is a primary potential risk factor for PF [14,17]. Hence, weakness of extrinsic foot muscles, such as the peroneus longus and gastrocnemius, may be considered as a possible contributing factor to PF [15,18] because they increase the stress on the plantar fascia [7,14,18]. However, no study has investigated the correlation between the performance of lower extremity muscles, such as the hip, quadriceps, hamstring, and ankle plantar flexor, and foot pressure among patients who have PF with and without a flat foot posture. In particular, investigating differences in lower extremity muscle performance and foot pressure in patients with PF with different foot postures is important to identify the etiology of PF, as changes in lower extremity biomechanics affect the plantar fascial load [7].

The purpose of this study was to analyze the differences in lower extremity muscle performance, such as muscle strength and reaction time, and foot pressure between patients who have PF with and without a flat foot posture, using a quantitative measurement device and pedobarography. We hypothesized that there would be lower muscle strength, a faster reaction time, and higher foot pressure in the involved ankles of patients who have PF with flat foot posture compared with those without flat foot posture.

## 2. Materials and Methods

### 2.1. Study Participants

**Figure 1:**
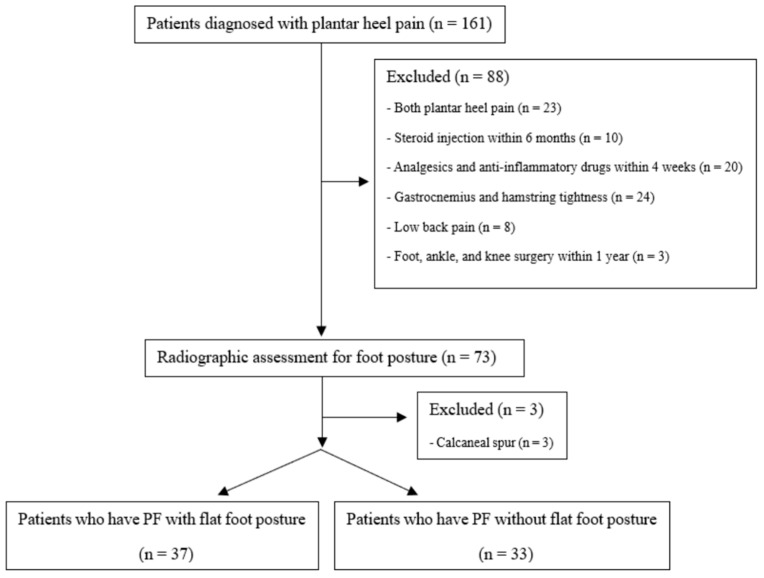
Flowchart of patients with plantar heel pain.

This study complied with the principles of the Declaration of Helsinki and was approved by the Institutional Review Board of our institute. The participants were recruited through medical consultations, and informed consent was obtained from all the patients and/or their legal guardians. All studies were performed in accordance with the relevant guidelines and regulations. This prospective observational study included 161 consecutive patients with plantar heel pain diagnosed between 2018 and 2020 using physical examination and plain radiography by two orthopedic surgeons. The main symptom was localized heel pain, and patients experienced the worst pain while taking their first steps in the morning or when walking after a period of rest. The inclusion criteria were patients who have PF with normal foot posture and those with abnormal foot posture, such as flat foot, which is defined as a talonavicular coverage angle > 7°, a lateral talo-first metatarsal angle > 5°, and a calcaneal inclination angle < 18° [10,11]. We excluded patients with plantar heel pain; calcaneal spur; injections in the past 6 months; administration of analgesics and anti-inflammatory drugs within 4 weeks; tightness of the gastrocnemius and hamstring muscles; differences in leg length; foot, ankle, and knee surgery within 1 year; and lower back pain with neurologic signs. We also confirmed the absence of a flat foot posture in the uninvolved feet of the patients in both groups. Ninety-one patients were excluded based on the above exclusion criteria. Thus, 70 patients (37 patients who have PF with flat feet vs. 33 patients who have PF with normal feet; Figure 1) were finally enrolled in the study.

### 2.2. Isometric Hip Muscle Strength

Based on a previous study [19], isometric hip muscle tests were performed in the side-lying position using a handheld digital dynamometer (Hoggan Health Industries, Inc., West Jordan, UT, USA), and pressure was applied approximately 5 cm above the lateral condyle of the femur. The duration of maximal isometric contraction was standardized at 5 s, with a resting time of 1 min for evaluation on the opposite side. The examiner recorded the average data twice, and muscle strength was normalized to the patient’s body weight (kgf/kg). The intraclass correlation coefficient (ICC) for the isometric hip muscle test in this study was 0.91.

### 2.3. Isokinetic Muscle Strength of the Quadriceps, Hamstring, and Plantar Flexor

The strength of the lower extremity muscles, including the quadriceps, hamstring, and plantar flexor muscles, was measured using an isokinetic device (Biodex Multi-Joint System 4, Biodex Medical Systems, Inc., Shirley, NY, USA) [9]. Strength of the quadriceps and hamstring muscles was measured with the patient in a seated position. Five submaximal knee flexion and extension motions at 180°/s were conducted for warm-up, followed by the testing, which included five maximal contractions for muscle strength at 180°/s. Knee flexion motion determined hamstring muscle function, and extension motion determined quadriceps muscle function. The plantar flexor muscle strength was evaluated for five maximal plantar flexion contractions at 120°/s in a semi-seated position with 20° knee flexion. Muscle strength was recorded as the peak torque normalized to the patient’s body weight (peak torque/body weight, Nmkg^−1^ × 100). In this study, the ICC was 0.83 for hamstring strength, 0.88 for quadriceps strength, and 0.81 for plantar flexor strength.

### 2.4. Isokinetic Muscle Reaction Times for Quadriceps, Hamstring, and Plantar Flexor

Isokinetic muscle reaction time was evaluated using acceleration time (AT), defined as the time (ms) taken to attain a preset angular velocity (180°/s for the knee joint and 120°/s for the ankle joint) during maximal muscle contraction [9,20,21]. Higher AT values indicate delayed muscle reaction time. The AT was automatically calculated using the Biodex software program during the muscle strength test. The ICC for the ATs was 0.87 for the hamstrings, 0.90 for the quadriceps, and 0.79 for the plantar flexor.

### 2.5. Foot Pressure

Foot pressure parameters, such as peak plantar pressure and pressure–time integrals, were measured using pedobarography (Tekscan, Inc., Boston, MA, USA). Based on previous studies [9,22], the peak pressure and pressure–time integrals were calculated for each of the five foot segments. Peak pressure is defined as the maximum pressure (KPa) in each of the three areas (forefoot, midfoot, and rearfoot) during gait. The pressure–time integral is defined as the time integral of the mean pressure (Ns) in each of the three areas (forefoot, midfoot, and rearfoot) during gait. Previous studies have shown that pressure–time integrals may be better indicators of foot function than peak pressure [23,24]. All patients practiced stepping on the pressure platform in three steps with the affected foot while walking for 2 m, followed by 3 times assessment. The ICC for the peak plantar pressure was 0.77.

### 2.6. Statistical Analysis

Based on a previous study on sample size calculation [9], a quadricep’s muscle difference of >10% between the groups was considered clinically significant. A priori power analysis (alpha level of 0.05, power of 0.8) was used to determine the sample size. From the results of a pilot study involving five ankles in each group, the effect size (Cohen’s d: 1.037) was calculated, and 16 ankles in each group were required to identify a clinically significant difference of >10% in the quadriceps muscle between the groups. The power of this study was 0.810. Student’s *t*-test was used to compare the performance of lower extremity muscles, including the hip, quadriceps, hamstring, and plantar flexor, and foot pressure parameters, including peak plantar pressure and pressure–time integrals, between patients who have PF with and without flat feet. A paired *t*-test was used to compare the two related variables between the involved and uninvolved feet of each patient in both groups. The Shapiro test was used to determine the normal distribution of continuous variables. Data were analyzed using SPSS version 17.0 (SPSS Inc., Chicago, IL, USA). *p* < 0.05 was considered statistically significant. All statistical analyses were performed by a statistician.

## 3. Results

Table 1 shows the demographic data of patients who have PF, with and without a flat-foot posture. There were no significant differences in age, sex, height, weight, body mass index, or visual analog scale score between the patients in both groups (*p* > 0.05).

### 3.1. Comparison of Muscle Strength between the Patient Groups

The strengths of the hip, quadriceps, and hamstring muscles were not significantly different between the groups (*p* > 0.05). However, the plantar flexor muscle strength was significantly lower in the involved ankles of patients who have PF with flat foot posture when compared with those without flat foot posture [39.1 ± 11.8 Nmkg^−1^ × 100 vs. 47.0 ± 12.4 Nmkg^−1^ × 100, 95% confidence interval (CI): −13.6 to −2.1, effect size: −0.652, *p* = 0.008; Table 2]. The strength of the hip, quadriceps, hamstring, and plantar flexor muscles in the uninvolved ankles did not differ between the patient groups (*p* > 0.05).

### 3.2. Comparison of Muscle Reaction Time between the Patient Groups

The ATs values of the quadriceps and hamstring muscles were not significantly different between the patient groups (*p* > 0.05). The plantar flexor AT was significantly lower in the involved ankles of patients who have PF with flat foot posture compared with those without flat foot posture (42.8 ± 16.6 ms vs. 53.6 ± 15.6 ms, 95% CI: −18.5 to −3.0, effect size: −0.670, *p* = 0.007, Table 2). In the uninvolved ankles, the ATs of the quadriceps, hamstring, and plantar flexor muscles showed no significant differences between the patient groups (*p* > 0.05).

### 3.3. Comparison of Foot Pressure between the Patient Groups

Peak plantar pressure and pressure–time integrals for each of the three areas (forefoot, midfoot, and rearfoot) were not significantly different between the patient groups (*p* > 0.05, Table 3).

### 3.4. Comparison of Lower Extremity Muscle Performance between Both Feet in Each Patient Group

**Figure 2:**
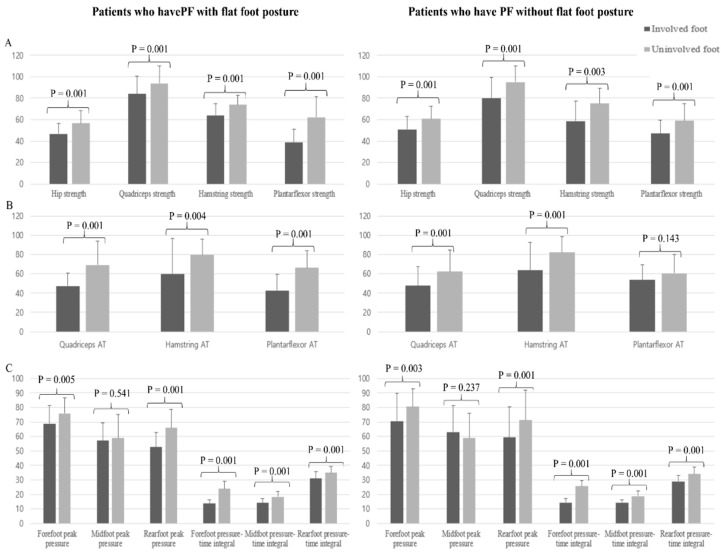
Lower extremity muscles performance [strength (**A**) and AT (**B**)] and foot pressure (**C**) in patients who have PF, with and without a flat foot posture. PF, plantar fasciitis; AT, acceleration time.

The strengths of the hip, quadriceps, hamstring, and plantar flexor muscles were significantly lower (*p* < 0.05, Figure 2A) in the involved foot than in the uninvolved foot in both groups. The ATs of the quadriceps, hamstring, and plantar flexor muscles were significantly faster (*p* < 0.05, Figure 2B) in the involved foot than in the uninvolved foot of patients in both groups, except for the ATs of the plantar flexor muscles of PF patients without flat foot posture. (*p* > 0.05, Figure 2B).

### 3.5. Comparison of Foot Pressure between Both Feet in Each Patient Group

The peak plantar pressure and pressure–time integrals for each of the three areas (forefoot, midfoot, and rearfoot) were significantly lower (*p* < 0.05, Figure 2C) in the involved foot than in the uninvolved foot of patients in both groups, but not the peak plantar pressure in the midfoot (*p* > 0.05, Figure 2C).

## 4. Discussion

The most important finding of this study was that the strength of the plantar flexor muscle was significantly decreased, whereas the reaction time of the plantar flexor muscle was significantly faster in the involved feet of PF patients with flat foot posture than in the foot of those without flat foot posture. Furthermore, performance deficits in the lower extremity muscles and different foot pressures were observed between the involved and uninvolved feet of patients in both groups.

A review [25] and a previous study [18] showed that plantar flexor muscle weakness is a strong factor causing PF. In this study, the hip, quadriceps, and hamstring muscle strengths were not different between the patient groups, whereas the plantar flexor muscle strength was significantly lower in the involved foot of PF patients with a flat foot posture. A possible explanation for this may be the weakening caused by overuse of the plantar flexor muscle for propulsion during gait. Sadeghi et al. [26], reported that plantar flexor muscles play a significant role in propulsion. However, patients who have PF with a flat foot posture have a reduced stability of the foot arch and ankle joint, resulting in ineffective propulsion [7,26]. Therefore, the plantar flexor muscle in patients who have PF with flat feet is overused for propulsion, and, consequently, weakening may occur. However, Lee et al. [9] found no difference in plantar flexor strength between patients who have PF with normal foot posture and healthy controls. Alternatively, in patients who have PF with a flat foot posture, the use of the plantar flexor muscles to reduce plantar fascia tension in daily life may have a negative impact as excessive stretching of the plantar fascia during gait aggravates plantar heel pain. Sullivan et al. [15] showed that patients who have PF with flat foot posture had a weakened peroneus longus compared with normal controls, which reduced plantar fascia loading by the flat foot during the late stance. The peroneus longus is highly active in plantar flexion during the late stance [27]. This may explain why patients who have PF with a flat foot posture had significantly lower plantar flexor strength than those without flat foot postures.

In this study, the ATs of the quadriceps and hamstrings were not significantly different between the patient groups, whereas the plantar flexor AT was significantly faster in the involved foot of PF patients with flat foot posture than in the feet of those without flat foot posture. Although the reasons for this are unclear, it may be due to compensatory mechanisms to improve the foot arch and postural stability during gait. A previous study [28] reported that postural stability was reduced in flat-arched feet due to foot hypermobility during weight bearing. Murley et al. [29]. investigated the electromyographic (EMG) data of the tibialis posterior muscle in 30 patients each in the flat foot posture and normal foot posture groups, and found that the EMG activity of the tibialis posterior muscle was increased in patients in the flat foot posture group. Thus, a neuromuscular compensation mechanism to promote foot stability in patients with flat foot posture has been reported [29]. The tibialis posterior is a plantar flexor muscle that plays an important role in stabilizing the foot by maintaining its arch. Furthermore, Petrofsky et al. [30]. reported a loss of dynamic balance in patients with PF. Plantar flexor muscle activity also plays an important role in maintaining body support against gravity [26]. Lee et al. [9]. reported faster plantar flexor muscle activity for postural stabilization in PF patients with normal feet than in healthy controls. These results possibly explain why patients who have PF with a flat foot posture achieved faster plantar flexion AT than those without a flat foot posture. In particular, the lack of difference in foot pressure results between the patient groups in this study may also be due to the rapid plantar flexor muscle reaction in patients with PF with flat foot posture contributing to foot [29] and postural stability [26]. Another possible reason for the results of this study may be the mechanism of the cerebral processing of pain. Ploner et al. [31] reported that cerebral organization of pain processing enhances motor responses to potentially harmful stimuli. Compared with patients with PF who have normal feet, patients with PF who have flat feet experience severe pain due to excessive stretching of the plantar fascia during walking; thus, the plantar flexor muscles may respond quickly as compensation to reduce pain. Further studies are necessary to validate the results of this study.

This study had some limitations. First, there were no normal controls; however, the contralateral uninvolved foot was used as the normal control for both patient groups. Second, patients with high-arched feet were excluded. These patients also have abnormal foot posture and are known to produce higher peak pressure on the heel while walking [7]. Therefore, further studies on lower extremity muscle performance in PF patients with high-arched feet are needed. Third, the differences in leg length between the two groups were not assessed. Mahmood et al. [32] reported that a longer leg length may be the cause of PF; thus, further studies are needed to clarify the results of this study. Fourth, changes in walking speed and gait could potentially affect foot pressure results; thus, examiners should be cautious when evaluating foot pressure. Fifth, intrinsic foot muscle strength was not measured. Previous studies have reported the significant involvement of intrinsic foot muscles, such as the abductor hallucis [33,34] and toe flexor [15,35,36], in maintaining foot arch stability [33,37]. Hence, the weakness of these muscles may have induced a rapid reaction of the plantar flexor muscle to improve foot and postural stability in patients who have PF with a flat foot posture. To confirm this, further evaluation of plantar flexor muscle function should be performed following rehabilitation for intrinsic and extrinsic foot muscles, and postural stability. Finally, we did not perform an EMG test to evaluate the muscle reaction time. In particular, the activation of the tibialis posterior muscle for foot stabilization mentioned in this study requires precise evaluation using EMG. Nevertheless, several studies have assessed AT using an isokinetic device to evaluate the reaction time of the plantar flexor muscles [20,21].

## 5. Conclusions

Patients who have PF with a flat foot posture showed decreased strength of the plantar flexor muscle compared with those without a flat foot posture, whereas patients who have PF without a flat foot posture showed a slower reaction time of the plantar flexor muscle compared with those with a flat foot posture. This study confirmed the differences in muscle performance between patients who have PF with different foot postures. Therefore, clinicians and therapists should plan treatment while considering the differences in these characteristics for the management of these patients.

## Figures and Tables

**Table 1:** Demographic data of enrolled patients who have PF with and without flat foot posture.

	Patients Who Have PF with Flat Foot Posture (*n* = 37)	Patients Who Have PF without Flat Foot Posture (*n* = 33)	*p*-Value
Sex (male/female)	21/16	19/14	0.945
Age (years) ^a^	51 ± 10.3	48 ± 8.9	0.214
Height (cm) ^a^	168 ± 8.0	166 ± 8.2	0.509
Weight (kg) ^a^	71.2 ± 10.5	69.4 ± 7.6	0.419
BMI (kg/m^2^) ^a^	24.8 ± 4.2	22.3 ± 3.0	0.590
VAS at activity	5.0 ± 0.9	4.8 ± 0.7	0.411
Foot pain side (right/left)	26/11	21/12	

PF, plantar fasciitis; BMI, body mass index; VAS, visual analogue scale. ^a^ The values are expressed as mean ± standard deviation.

**Table 2:** Comparison of muscle strength and AT in both ankles of patients who have PF with and without flat foot posture.

	Involved Foot			Uninvolved Foot		
	Patients Who Have PF with Flat Foot Posture	Patients Who Have PF without Flat Foot Posture	*p*-Value	Patients Who Have PF with Flat Foot Posture	Patients Who Have PF without Flat Foot Posture	*p*-Value
Hip strength	46.9 ± 9.3	51.0 ± 12.1	0.119	56.7 ± 11.8	60.9 ± 11.6	0.140
MD, (95% CI)	−4.5, (−9.1, 1.0)			−4.2, (−9.8, 1.4)		
Effect size	−0.379			−0.358		
Quadriceps strength	84.1 ± 16.6	80.2 ± 19.3	0.363	93.7 ± 16.6	95.0 ± 14.9	0.726
MD, (95% CI)	3.9, (−4.6, 12.5)			−1.3, (−8.9, 6.2)		
Effect size	0.216			−0.082		
Hamstring strength	64.1 ± 11.0	58.8 ± 18.7	0.152	73.9 ± 8.7	75.1 ± 14.3	0.668
MD, (95% CI)	5.2, (−1.9, 12.5)			−1.2, (−6.8, 4.4)		
Effect size	0.345			−0.101		
Plantar flexor strength	39.1 ± 11.8	47.0 ± 12.4	0.008 ^a^	62.1 ± 19.2	59.0 ± 16.2	0.162
MD, (95% CI)	−7.9, (−13.6, −2.1)			3.1, (−6.4, 10.6)		
Effect size	−0.652			0.174		
Quadriceps AT	47.2 ± 13.8	47.8 ± 19.8	0.886	69.4 ± 24.4	62.4 ± 22.3	0.216
MD, (95% CI)	−0.5, (−8.6, 7.5)			7.0, (−4.1, 18.2)		
Effect size	−0.035			0.299		
Hamstring AT	79.4 ± 36.8	82.1 ± 29.3	0.741	60.0 ± 16.8	63.6 ± 16.5	0.366
MD, (95% CI)	−2.6, (−18.9, 13.3)			−3.6, (−11.6, 4.3)		
Effect size	−0.081			−0.216		
Plantar flexor AT	42.8 ± 16.6	53.6 ± 15.6	0.007 ^a^	66.4 ± 17.6	60.6 ± 19.2	0.195
MD, (95% CI)	−10.7, (−18.5, −3.0)			5.7, (−3.0, 14.5)		
Effect size	−0.670			0.314		

PF, plantar fasciitis, MD, Mean difference; CI, Confidence interval; AT, acceleration time. Note: The values are expressed as mean ± standard deviation. The measurement unit for muscle strength was Nmkg^−1^ × 100 and reaction time was ms. ^a^ Bold means statistically significant.

**Table 3:** Comparison of foot pressure in both feet of the patients who have PF with and without flat foot posture.

	Involved Foot			Uninvolved Foot		
	Patients Who Have PF with Flat Foot Posture	Patients Who Have PF without Flat Foot Posture	*p*-Value	Patients Who Have PF with Flat Foot Posture	Patients Who Have PF without Flat Foot Posture	*p*-Value
Forefoot peak pressure	68.8 ± 12.4	70.6 ± 19.3	0.634	75.9 ± 10.5	80.6 ± 12.2	0.091
MD, (95% CI)	−1.8, (−9.5, 5.8)			−4.6, (−10.1, 0.7)		
Effect size	−0.110			−0.412		
Midfoot peak pressure	57.0 ± 12.4	63.1 ± 18.2	0.102	58.8 ± 16.2	58.9 ± 16.9	0.980
MD, (95% CI)	−6.1, (−13.5, 1.2)			−0.1, (−8.0, 7.8)		
Effect size	−0.391			−0.006		
Rearfoot peak pressure	52.6 ± 10.0	59.3 ± 21.0	0.086	65.9 ± 12.8	71.2 ± 20.9	0.200
MD, (95% CI)	−6.7, (−14.5, 0.9)			−5.3, (−13.5, 2.8)		
Effect size	−0.407			−0.305		
Forefoot pressure–time integral	13.8 ± 2.5	14.4 ± 2.6	0.288	24.0 ± 5.1	25.6 ± 3.9	0.159
MD, (95% CI)	−0.6, (−1.8, 0.5)			−1.6, (−3.8, 0.6)		
Effect size	−0.235			−0.352		
Midfoot pressure–time integral	14.3 ± 2.8	14.5 ± 1.9	0.785	18.5 ± 3.4	18.9 ± 3.6	0.632
MD, (95% CI)	−1.2, (−1.3, 1.2)			−0.4, (−2.1, 1.2)		
Effect size	−0.083			−0.114		
Rearfoot pressure–time integral	30.9 ± 4.9	29.0 ± 4.0	0.087	34.9 ± 4.2	34.1 ± 4.6	0.492
MD, (95% CI)	1.9, (−0.2, 4.0)			0.8, (−1.3, 2.8)		
Effect size	0.424			0.181		

PF, plantar fasciitis; MD, mean difference; CI, confidence interval. Note: The values are expressed as mean ± standard deviation. The measurement units are KPa for the peak pressure and Ns for the pressure–time integral.

## Data Availability

The data presented in this study are available upon request from the corresponding author.
